# Prevalence of HCV among the young male blood donors of Quetta region of Balochistan, Pakistan

**DOI:** 10.1186/1743-422X-10-83

**Published:** 2013-03-13

**Authors:** Ayesha Khan, Abdul Malik Tareen, Aamer Ikram, Hazir Rahman, Abdul Wadood, Muhammad Qasim, Kalimullah Khan

**Affiliations:** 1 Department of Microbiology, University of Balochistan, Quetta, Pakistan; 2 Department of Microbiology, Kohat University of Science and Technology, Kohat, Khyber Pakhtunkhwa, 26000, Pakistan; 3 Combined Military Hospital, Quetta, Pakistan

**Keywords:** Enzyme linked immunosorbant assay, Hepatitis C virus, Immunochromatography, Quetta, Pakistan

## Abstract

**Background:**

Hepatitis C, caused by hepatitis C virus (HCV) is a contagious disease of the liver which infects more than 170 million people world-wide and around 16 million in Pakistan. HCV associated infection spreads mainly by blood-to-blood contact. In recent years, many studies have been conducted to determine the prevalence of HCV infection in Pakistan; however, no data is available on HCV infection from the largest province of Pakistan. Therefore, the present study focuses on the prevalence of HCV infection in the young male blood donor population of Quetta region of Balochistan, Pakistan.

**Methods:**

A total of 356 blood samples were collected from blood donors (age range 17–25 years) at Combined Military Hospital (CMH), Quetta, Balochistan, Pakistan. Blood samples were screened for HCV positivity by Immunochromatographic test (ICT) and Enzyme Linked Immunosorbant Assay (ELISA).

**Results:**

Out of 356 blood samples, the overall HCV prevalence was 20.8%. Among the HCV positive cases, the age group with 25 years was more frequently infected with a prevalence of 26.3%.

**Conclusions:**

The present study provides the preliminary information about high HCV prevalence among the young male donor population in Balochistan province. This data may be helpful in formulating public health strategy for the prevention of risk factors associated with spreading of the disease. Furthermore, we recommend that in public sector hospitals and health care units ELISA should be preferred for anti-HCV detection over ICT.

## Background

Hepatitis C is an infectious disease caused by the hepatitis C virus (HCV) primarily affecting the liver [1]. HCV has a single-stranded RNA genome and belongs to Hepacivirus [2]. Infected blood, blood products and body fluid are some major risk factors for HCV transmission. Furthermore, use of contaminated syringes, drug abuse, and use of barber razor, dental procedures, tattooing, ear piercing, acupuncture and high-risk sexual behavior are other modes of transmission [3]. In more than 70% of the infected people, the disease becomes chronic and leads to chronic hepatitis, 5-20% develops cirrhosis, and 1–5% died from cirrhosis or liver cancer [4]. Recently, World Health Organization (WHO) reported that nearly 170 million people are chronically infected with hepatitis C virus worldwide. In Asia-pacific region, the prevalence of chronic hepatitis C ranges from 4% to 12% [5]. In Pakistan more than 10 million people are suffering from HCV that comprise to 6% of total population of Pakistan, with high morbidity and mortality [6]. In previous small studies HCV prevalence in some other cities of Pakistan was reported high as well. Like it was 16% in Lahore, 20.6% in Faisalabad and 23.8% in Gujranwala [7,8].

The HCV diagnosis is carried out by detecting the presence of circulating anti-HCV antibodies using Immunochromatographic test (ICT) methods; however due the false positivity rate of HCV with ICT based methods, Enzyme Linked Immunosorbant Assay (ELISA) is considered more reliable than ICT based HCV diagnosis. Previously, a small scale study was conducted reporting the prevalence of HCV genotypes in the general population of Balochistan province of Pakistan [9] and Hepatitis C and B virus seroprevalence and coinfection along with HIV in injecting drug users of Quetta region of Balochistan [10]. However, a general screening study for HCV infection in the young healthy male population of the province was not yet reported. Therefore, the present study was undertaken to find the prevalence of HCV infection in the young males ranging from 17 to 25 years in Balochistan, Pakistan. Moreover, use of an ICT (Immuno-chromatographic test) and ELISA (Enzyme-Linked Immunosorbent Assay) coupled HCV screening approaches were used to report the HCV prevalence. These findings may be helpful to devise strategy for the prevention of HCV associated risk factors.

## Results

### HCV prevalence among the young blood donors of Quetta, Balochistan

A total of 356 blood samples were taken from healthy young male blood donor population with mean age 21(±4) years. Total numbers of samples in different age groups are listed in Table 1. Of total 356 samples, 79 (22.2%) were positive for anti-HCV antibodies by ICT method (Table 1). Furthermore, positive samples were confirmed by ELISA. In the ICT positive samples 79 (22.2%), 74 (20.8%) samples were positive for anti-HCV antibodies using ELISA method (Table 1).

**Table 1:** Frequency of HCV infection according to age groups among the young blood donors of Quetta, Balochistan

**Age group (years)**	**Sample size (n)**	**Positive anti-HCV (by ICT)**	**Percentage (%)**	**Positive anti-HCV (by ELISA)**	**Percentage (%)**
17	23	5	21.7	5	21.7
18	21	4	19.0	4	19.0
19	44	6	13.6	6	13.6
20	49	13	26.5	12	24.5
21	54	11	20.4	10	18.5
22	40	7	17.5	7	17.5
23	33	8	24.2	8	24.2
24	54	14	25.9	12	22.2
25	38	11	28.9	10	26.3
Total	**356**	**79**	**22.2%**	**74**	**20.8%**

## Discussion

Hepatitis C is a global health problem and every year up to 170 million people become infected with HCV worldwide with a mortality rate of more than 350,000 people [5]. HCV has high prevalence in developing and also high populated countries including Pakistan. Several factors contribute to increasing HCV prevalence such as lack of awareness regarding HCV transmission, and precautionary measures among common people, inadequate diagnostic facilities and expertise in hospitals and public sector laboratories [3].

In recent years, many studies have been conducted to investigate the prevalence of HCV in Pakistan [6,11]. As, only a few (20-50%) of infected individuals clear the virus spontaneously, while the majority of patients develop chronic hepatitis that is why a high alarming frequency of hepatitis c viral infection was seen in different parts of the country, such as 20.6% in Faisalabad and 23.8% in Gujranwala [7,8]. Previously, a study was conducted to determine the prevalence of HCV genotypes in the general population of Balochistan province of Pakistan [9]. Furthermore, Hepatitis C and B virus seroprevalence and co-infections along with HIV in injecting drug users of Quetta were also reported [10].

In the present study commonly practiced serological techniques (ICT and ELISA) were employed to screen out the prevalence of HCV in the young blood donors in age group 17–25 years (Table 1). We found that overall prevalence of HCV was 22.2% and 20.2% when tested by ICT and ELISA based methods respectively. Interestingly we observed that maximum prevalence (26.3%) for anti-HCV was found in the individuals of 25 years old (n = 38) (Table 1). Though our study provides no information regarding HCV incidence in the general population; however it does represent the HCV prevalence in the young blood donors, which reflects a more specific study. The highest burden of HCV in young individuals were observed in Quetta region of Baluchistan using ICT and ELISA based HCV diagnostics which are not routinely done in other hospitals of Baloschistan. This might be further evaluated in increased sample size, and PCR based HCV diagnostics in young blood donors.

In general practice, it was consistently reported that screening by ICT method may produce false positive results, and is not recommended as a single diagnostic tool for HCV detection [3,12]. To minimize the chances of false positivity, we rechecked all the positive samples checked by ICT method, were also subjected to screen by using third generation ELISA. These finding suggest that considering ICT as diagnostic tool might be misleading in some cases so critical care must be taken in account before reporting the results. Our results indicated that one sample each in the age group of 20, 21 and 25 years was false positive for anti-HCV. Moreover, two samples in the age group of 24 years old were confirmed false positive on ELISA. Hence, the anti-HCV prevalence dropped from 28.9% to 26.3% in the age group 25 year old, from 26.5% to 24.5% in the age group 20 year old, from 20.4% to 18.5% in the age group of 21 year old and from 25.9% to 22.2% in a the age group of 24 years old respectively.

Results of the present study show high HCV prevalence in the young population of Quetta region because Pakistan still remains a high prone zone for HCV infection. In addition, Ahmed et al. 2007 and Aslam M et al. 2007 previously reported high HCV prevalence (up to 23.8%) in people of other parts of Pakistan. Furthermore, the geographic location of Quetta (on border with Afghanistan) and large influx of Afghan refugees contribute also to high HCV infection. Poor health facilities and infection control in public hospitals and lack of healthcare-related awareness are some of the other risk factors contributing to the high HCV prevalence in this region. As the present study is comprised of less number of participants who visited a hospital and were screened positive for HCV. It might be possible that participants of this study were infected already but they were not gone through medical examination before this. Our data also indicates that testing of blood and blood products by ICT devices may not be used as a single reliable tool for the anti-HCV prevalence, and may produce misleading findings. Interestingly, in Balochistan HCV screening is usually performed with ICT method only, so we suggest that ICT results should be reconfirmed by more sophisticated techniques such as ELISA and PCR in the hospitals and in all health care units. Although, the present study is not on large scale, but it will provide the preliminary information regarding the prevalence of HCV in the young blood donors of Quetta region of Balochistan.

## Conclusion

The present study highlights the preliminary information regarding the prevalence of HCV in the young population of Quetta, Balochistan. Furthermore, screening of HCV by ICT method seems less reliable and should be reconfirmed with ELISA and PCR method on parallel to improve the diagnostic tools for HCV in this region. The need is that the government should address this major health issue comprehensively by enhancing surveillance, prevention, care, and treatment.

## Materials and methods

### Study area and sample collection

Volunteer blood donors were included in this study from different areas of Balochistan mainly from Quetta and surrounding region. The study was conducted on those volunteer who reporting during the study period (January 2011 to April 2011) at the Combined Military Hospital (CMH) Quetta, Balochistan, Pakistan. The study was approved by the ethical committee of the University of Balochistan with reference number (EC/UOB/0094). Blood samples were collected from a total of 356 healthy young volunteer blood donors with an age range of 17–25 years upon their written consent. HCV screening was performed using ICT and ELISA based methods.

### Immuno-chromatographic tests (ICT)

Blood samples were collected in EDTA-tubes. Serum was isolated from blood samples for viral antibodies detection. anti-HCV antibodies was detected by ICT as described by manufacturers. For this purpose, immune-chromatographic strips were obtained from two different vendors including Accurate and Acon (Acon, USA).

### Enzyme linked immunosorbant assay (ELISA)

Positive sera confirmed though ICT were tested for anti-HCV antibodies by ELISA using Biokit, S.A, (Barcelona-Spain) according to the manufacturer’s protocol.

### Data analysis

The data was analyzed with Window 7, Microsoft Excel 2007 (Microsoft, USA). Hepatitis C incidence was calculated in different age groups in terms of percentages.

## Competing interests

The authors have declared that no competing interests exist.

## Authors’ contribution

AMT and KK designed the study and prepared the manuscript. AK and AI carried out the experimental work and AI also advised about protocol. AW, MQ and HZ helped in manuscript writing and critically reviewed the manuscript. All authors read and approved the final manuscript.
